# Strategies of tree species to adapt to drought from leaf stomatal regulation and stem embolism resistance to root properties

**DOI:** 10.3389/fpls.2022.926535

**Published:** 2022-09-27

**Authors:** Zhicheng Chen, Shan Li, Xianchong Wan, Shirong Liu

**Affiliations:** ^1^ Key Laboratory of Forest Ecology and Environment of National Forestry and Grassland Administration, Ecology and Nature Conservation Institute, Chinese Academy of Forestry, Beijing, China; ^2^ Department of Environmental Science and Ecology, School of Environmental Science and Engineering, Shaanxi University of Science and Technology, Xi’an, China; ^3^ Institute of Ecological Conservation and Restoration, Chinese Academy of Forestry, Beijing, China

**Keywords:** climate change, hydraulic traits, hydraulic failure, carbon starvation, stomatal safety margin, tree mortality

## Abstract

Considerable evidences highlight the occurrence of increasing widespread tree mortality as a result of global climate change-associated droughts. However, knowledge about the mechanisms underlying divergent strategies of various tree species to adapt to drought has remained remarkably insufficient. Leaf stomatal regulation and embolism resistance of stem xylem serves as two important strategies for tree species to prevent hydraulic failure and carbon starvation, as comprising interconnected physiological mechanisms underlying drought-induced tree mortality. Hence, the physiological and anatomical determinants of leaf stomatal regulation and stems xylem embolism resistance are evaluated and discussed. In addition, root properties related to drought tolerance are also reviewed. Species with greater investment in leaves and stems tend to maintain stomatal opening and resist stem embolism under drought conditions. The coordination between stomatal regulation and stem embolism resistance are summarized and discussed. Previous studies showed that hydraulic safety margin (HSM, the difference between minimum water potential and that causing xylem dysfunction) is a significant predictor of tree species mortality under drought conditions. Compared with HSM, stomatal safety margin (the difference between water potential at stomatal closure and that causing xylem dysfunction) more directly merge stomatal regulation strategies with xylem hydraulic strategies, illustrating a comprehensive framework to characterize plant response to drought. A combination of plant traits reflecting species’ response and adaptation to drought should be established in the future, and we propose four specific urgent issues as future research priorities.

## Introduction

Forests, as the dominant biomes of the global land, plays a crucial role in the biogeochemical cycle (Pan et al., 2011). A great amount of evidences highlights increasing forest mortality as a result of global climate change-associated severe drought, and such mortality can lead to large-scale detrimental effect on ecosystem structure and function, causing a conversion from the forests carbon sink into carbon source (Phillips et al., 2009; Hartmann et al., 2018). Global observations of drought-induced forest dieback have stressed a need to understand and predict the vulnerability of trees to more frequent drought in the future.

The mechanisms underlying divergent strategies of tree species to drought have remained remarkably difficult to study, leading to some uncertainty in forecasting the future of forests in the world (Arend et al., 2021). Moreover, differences among tree species in drought adaptability are also integral determinants of distributions and the probability of extinctions in the future (Bonan, 2008). At present, two interconnected physiological reasons underlying drought-induced tree death have evolved, hydraulic failure and carbon starvation (McDowell et al., 2008; McDowell, 2011; Choat et al., 2018; Brodribb et al., 2020).

Based on the cohesion-tension theory, water in plants is transported from the soil through the plant to the crown along a gradient of negative pressure (tension) in the conduits (tracheids or vessels) of the xylem (McDowell et al., 2008; Trugman et al., 2021). Nevertheless, during transpiration, embolism can occur when the pressure in the conduits becomes negative enough to cause air entry through pits into neighboring conduits (Tyree and Zimmermann, 2002). The conduit will become incapable of water transport if embolism occurs (Tyree and Zimmermann, 2002). The hydraulic failure is that drought-induced xylem embolism stops water flow, and subsequently plant tissues desiccate (McDowell et al., 2008). Therefore, tree’ xylem embolism resistance serves as an important strategy to prevent hydraulic failure (Pivovaroff et al., 2016). Another important hydraulic strategy for trees to cope with drought is the closure of stomata. The CO_2_ used for photosynthesis and the water lost in transpiration share the common pathway—the stomatal pores on leaf surfaces, and reduced transpiration through closuring stomata is inevitably accompanied by the expense of carbon gain. The term “carbon starvation” is that drought induced-stomatal closure (even leaf shedding) causes carbohydrates supply to drop off and the continued carbohydrates metabolism or even the impaired phloem conductance leads to plant tissues starve (McDowell et al., 2008; McDowell, 2011).

Therefore, xylem embolism resistance and stomatal regulation are closely related to hydraulic failure and carbon starvation. In other words, the risk of tree mortality is generally determined by the lethal thresholds of carbon depletion and/or hydraulic failure to which plants are exposed during drought. The lethal thresholds are mainly the thresholds of embolism resistance and stomatal regulation (McDowell et al., 2008; Choat et al., 2018; Blackman et al., 2019; Brodribb et al., 2020). In fact, previous studies have indeed shown that drought-induced mortality of a given species can be predicted by hydraulic thresholds (xylem pressure at which 50% loss of hydraulic conductance, P_50_, a hydraulic trait widely used to estimate hydraulic safety) and declining carbon availability (Choat et al., 2012; Anderegg et al., 2016; Anderegg et al., 2019 Chen et al., 2019a).

The water is limited in the soil, and the ability of terrestrial plants to resist embolism and regulate stoma is limited. A conifer species from arid regions of Western Australia, *Callitris tuberculata*, is the most embolism resistant tree species in the world to date (P_50_ = -18.8 MPa) (Larter et al., 2015). In addition, stomatal regulation is also a requisite condition for land plants evolved to adapt to the terrestrial environment and it is impossible that endless water from the soil to be transpired into the atmosphere. Therefore, tree species with different adaptation strategies are screened and survived in different environments, which also achieves colorful biodiversity in terrestrial environments with various moisture conditions.

In this review, we evaluate and discuss the physiological and anatomical determinant of xylem embolism resistance and leaf stomatal regulation. The coordination and trade-offs of the two drought adaptation strategies are also summarized. Root, as important organ for absorbing water and nutrients, and its coordination with leaf and stem are also reviewed. In addition, research prospects of trees’ drought adaptation strategies in the future are proposed.

## Stomatal regulation

Based on fossil record, stomata appear approximately 400 million years ago (Edwards et al., 1998; Brodribb and Holbrook, 2003). The first published stomatal measurement was about stomatal density (Sack and Buckley, 2016), and the recognition that stomata open and close by changes in guard cell turgor was in 1938 (Heath, 1938). Although the total area of stomatal pores occupies only a small fraction of the leaf surface, typically less than 3%, 98% of H_2_O and CO_2_ passes through these pores (Lawson and Blatt, 2014). The evolution of stoma is believed to be caused by the selective pressure of optimizing the ratio of CO_2_ uptake to water lost during gas exchange (Raven, 2002; Brodribb and Holbrook, 2003). When plants experience drought, stomatal closure will be the first response of plant to reduce transpiration (Bartlett et al., 2016).

Stomal opening or closing is controlled by inflating or deflating stomatal guard cells driven by influx or efflux of water, whose water potential is associated with chemical osmolytes or signals (such as pH, ABA, H^+^, K^+^, Cl_2_ and ethephon, etc.) and leaf water potential (Brodribb et al., 2014; Medeiros et al., 2015; Papanatsiou et al., 2019). Therefore, water potential plays a key role in determining stomatal movements. In addition, guard cell size (and geometry) also tends to affect the speed of stomatal movement, and larger stomata often exhibit slower responses (Lawson and Blatt, 2014). In this review, the summarization and discussion will focus on the effect of water status on stomatal opening and closure.

### The concept of isohydry and anisohydry

Significant variation in sensitivity and responsiveness of the opening and closing of stomata to external environmental and internal signaling cues to exist among species (Lawson and Blatt, 2014). Further, the species-specific differences in the relationship between stomatal behavior and dynamic variation in water conditions are important for understanding the species-specific differences in the performance of response and adaptation to the environment (Meinzer et al., 2016). Frequently, across species, stomatal regulation is classified along a continuum from isohydry to anisohydry (Brodribb and McAdam, 2013; Brodribb et al., 2014; Skelton et al., 2015; Meinzer et al., 2016; Chen et al., 2021b; Hartmann et al., 2021). This classification existed before the water potential concept (Slatyer and Taylor, 1960; Scholander et al., 1965) and initially focused more on the regulation of transpiration than on the maintaining stable leaf water potential (Ψ_leaf_) per se (Martínez-Vilalta and Garcia-Forner, 2017). Now the definition of iso/anisohydric is usually based on Ψ_leaf_, and anisohydric species is generally attributed to less stomatal sensitivity, allowing large variation in Ψ_leaf_; isohydric species typically exhibit strong stomatal regulation, leading to relatively stable Ψ_leaf_ (Tardieu and Simonneau, 1998; Klein, 2014; Meinzer et al., 2017).

### The quantification of iso/anisohydric behavior

Three methods are generally adopted for the quantification of iso/anisohydric behavior: (1) the relationship between stomatal conductance (G_s_) and Ψ_leaf_; (2) the relationship between predawn water potential (Ψ_pd_) and midday water potential (Ψ_md_); (3) the “hydroscape” method.

The quantification of iso/anisohydric behavior by definition refers to ascertain the sustainability of G_s_ in the decrease of Ψ_leaf_ during drought, so the water potential at stomatal closure (Ψ_close_) obtained by the G_s_-Ψ_leaf_ curve is a suitably quantitative indicator of iso/anisohydric behavior (Brodribb and Holbrook, 2003; Skelton et al., 2015; Chen et al., 2021b; Bartlett and Sinclair, 2021a). This G_s_-Ψ_leaf_ curve method need to measure a wide enough range of soil moisture or water potential and relevant G_s_ to construct intact curve for obtaining accurate Ψ_close_. In addition, G_s_ is also influenced by CO_2_ concentration, illumination, air temperature, etc., so these interference factors on G_s_ must be excluded as much as possible during the measurement. By analyzing Ψ_close_, many studies found that species-specific Ψ_close_ formed a continuum, not simply dichotomy between isohydric and anisohydric (Klein, 2014; Martin-StPaul et al., 2017; Pivovaroff et al., 2018; Chen et al., 2019a; Henry et al., 2019). In a study of 20 co-occurring temperate broadleaf tree species, Ψ_close_ was found to be in the range from -0.655 MPa to -5.54 MPa (Chen et al., 2019a). In a meta-analysis study gathered data from more than 100 species in different biomes, Ψ_close_ varied from -1 to -4.3 MPa (Martin-StPaul et al., 2017). Noticeably, in a study conducted in southern California with a Mediterranean-type climate, Ψ_close_ was found to be about -10 MPa in a chaparral species (Pivovaroff et al., 2018).

More isohydric species show less variability in Ψ_md_, but Ψ_md_ of anisohydric tree species present oppositely (Martínez-Vilalta and Garcia-Forner, 2017). Based on this definition, Martínez-Vilalta et al. (2014) classified the stomatal regulation behaviors of plants by the decline range of Ψ_md_ with the decrease of Ψ_pd_, i.e., the slope (σ) of the model. However, σ vary with soil water potential, which highlights that confirming iso/anisohydric regulation on the basis of differences between predawn and midday water potential is problematic (Hochberg et al., 2018). Furthermore, changes in Ψ_md_ and Ψ_pd_ are also affected by hydraulic conductance and G_s_, and the reductions in both have the opposite effects on Ψ_leaf_ (Martínez-Vilalta and Garcia-Forner, 2017). Thus, considering the large effect of the environment, for proper the degree of iso-anisohydry of the species’ genotypic effect, basic physiological characteristics (such as Ψ_close_ or its proxy, turgor loss point) should be adopted rather than the degree of relationship between Ψ_pd_ and Ψ_md_ (Hochberg et al., 2018).

Meinzer et al. (2016) coined a new metric, the “hydroscape”, to define tree species’ isohydricity. The hydroscape is a two-dimensional metric, defined as the area enclosed by the Ψ_pd_ and Ψ_md_ regression lines, and species with larger hydroscapes are more anisohydric. The hydroscape integrates more comprehensive information and more accurately quantifies the iso/anisohydric degree than simple metrics such as σ (Meinzer et al., 2016). Furthermore, the strong correlation between hydroscape and hydraulic traits also suggests that the hydroscape may provide more information about overall drought tolerance than metrics involving the rate of change in Ψ_md_ as Ψ_pd_ declines (Meinzer et al., 2016; Li et al., 2019).

**Figure 1:**
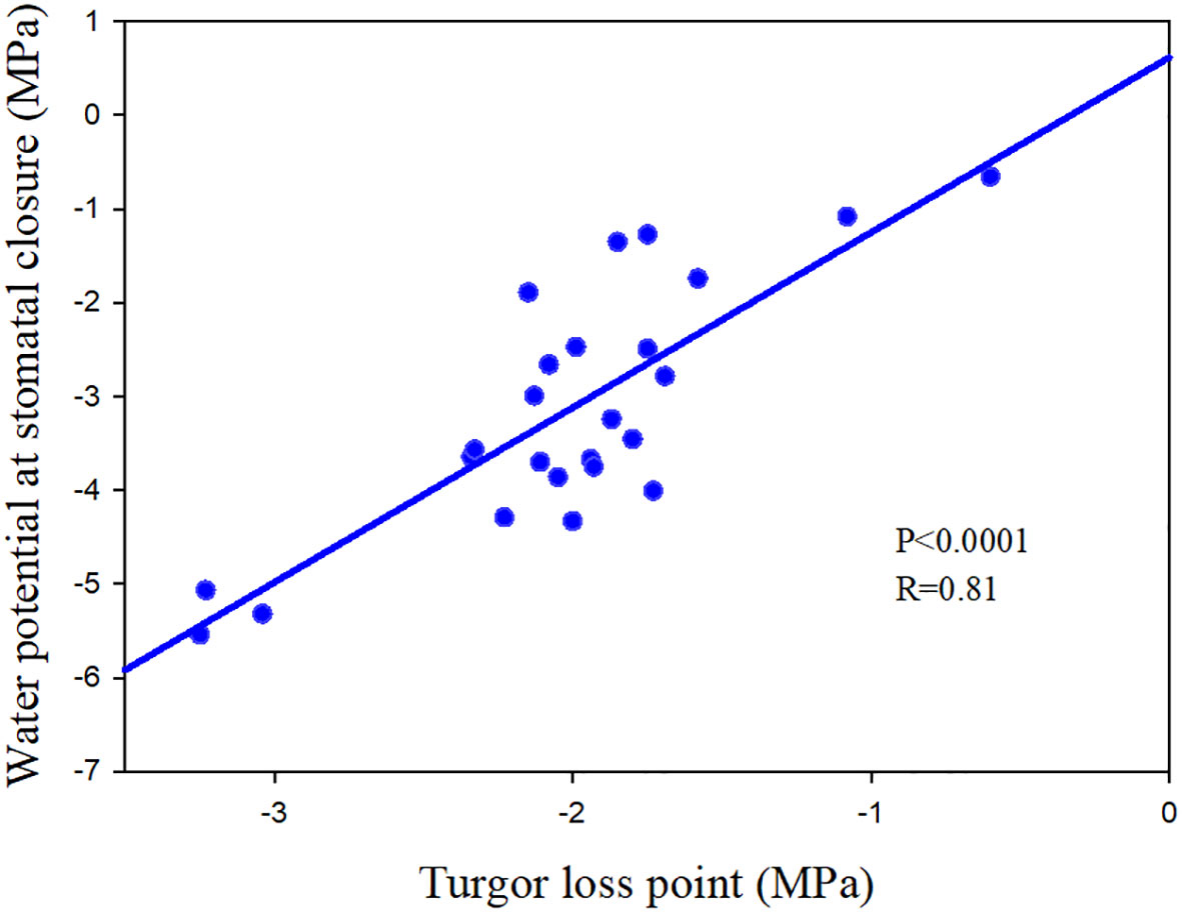
The relationship between turgor loss point and the water potential at stomatal closure of 25 angiosperm speices. The data comes from Chen et al. (2021b) and Li et al. (2015).

In addition to the three mentioned main methods, turgor loss point (TLP) was also regarded as a potential proxy for species stomatal regulation strategy (Hochberg et al., 2018), which is directly and closely linked with stomatal regulation (Brodribb and Holbrook, 2003; Chen et al., 2021b; **Figure 1**).

### The determinants of iso/anisohydric behavior

Regardless of how iso/anisohydric behavior is defined, it has been shown to be associated with a variety of stem and leaf traits (Fu et al., 2019; Chen et al., 2021b). The determinants or relevant traits of stomatal behavior are mainly phytohormone ABA, hydraulic traits and structural investment as discussed below.

#### ABA

ABA can cause a reduction in guard cell turgor, subsequently closing stomata (Brodribb and McAdam, 2013). However, the earliest stomatal response was an passive-hydraulic regulation without the requirement of ABA. This can be found in the extant species of basal vascular plants, including the lycophytes, ferns, and gymnosperms in evolutionary terms (McAdam and Brodribb, 2015). The passive-hydraulic regulation in these species show that stomata are like valves closing or opening in response to, respectively, a decrease or increase in turgor (McAdam and Brodribb, 2015).

The traditional studies suggest that ABA is a root-related hormone which is transported to the foliage through the stem xylem (Davies and Zhang, 1991), but short-term responses of ABA cannot be made clear by xylem transport (Lee et al., 2006). Now mounting molecular evidence illustrates the capacity of foliage and even guard cells to generate ABA (Bauer et al., 2013), and the ABA concentration may increase after short-term exposure of foliage to drought (Waadt et al., 2014; McAdam and Brodribb, 2015). In addition, the vascular tissues, including the phloem cells, can also synthesize ABA and transport ABA to the guard cells (Okamoto et al., 2009; Kuromori et al., 2014).

Tardieu and Simonneau (1998) proposed that qualitative differences in ABA may be the source of the differences between anisohydric and isohydric plants. Among conifers, contrasting iso/anisohydric behaviors were found to be related to divergent trends in xylem embolism resistance and foliar ABA dynamics during drought, with a functional phylogeny type (Brodribb et al., 2014). The rise in ABA in the xylem or leaf can activate anion channels, causing a reduction of guard cell turgor and subsequent stomatal closure. This mode fits with some species, whose stomata were sensitive to ABA as drought stress intensified, but not fit with other species (Brodribb and McAdam, 2013).

The physiological evidences from 42 species of conifers showed that the evolutionary mechanism of stomatal regulation of gymnosperm species follows two distinct pathways (Brodribb et al., 2014). In gymnosperm species with isohydric behavior, foliar ABA levels rapidly rise during drought stress exposure, and high levels of foliar ABA induce complete stomatal closure at relatively high water potential (ABA-rising type) (Brodribb et al., 2014; Kollist et al., 2014). However, in some gymnosperm species with anisohydric behavior, stomatal initial closure is induced by increased ABA and then by a transition to water potential during drought, and the stomata could only be completely closed when the negative water potential pull the guard cell turgor low enough (Brodribb and McAdam, 2013; Brodribb et al., 2014). The similar results have been also obtained in angiosperms, and thus the relationship between ABA dynamics and iso/anisohydric behaviors may be commonplace in woody plants (Brodribb and McAdam, 2013; Nolan et al., 2017). Given that ABA is likely to be an important determinant of stomatal regulation type, the iso/anisohydric behaviors may be regulated by relatively few genes, namely those regulating ABA catabolism or synthesis (Brodribb and McAdam, 2013; Brodribb and McAdam, 2015).

#### Hydraulic traits

Around the plant water use strategy of maximizing carbon gain and minimizing water loss, there are a set of hydraulic traits coordinated with iso/anisohydric behavior, which is not only the consequence of hydraulic regulation but also related to costs and gains.

Stomatal behavior directly affects plant water potential, and in previous research minimum midday water potential was strongly correlated with Ψ_close_, suggesting that Ψ_close_ crucially affects water stress the leaves experience (Bartlett et al., 2016). TLP is an important trait reflecting drought stress tolerance of tree species (Zhu et al., 2018), and stomatal behavior is largely determined by leaf turgor (**Figure 1**). Lenz et al. (2006) found that TLP was more negative in species inhabited in lower rainfall sites and whose diurnal fluctuations in water potential were larger. Other leaf pressure–volume (P-V) traits, such as relative water content at TLP (RWC_tlp_) and osmotic potential at full turgor (Ψ_100_), were found to be strongly related to TLP and consequentially Ψ_close_. Meinzer et al. (2016) demonstrated that TLP and Ψ_100_ could serve as robust proxies for a species’ location along the iso/anisohydry continuum within a diverse group of woody species studied under similar conditions. Estimates of TLP and Ψ_100_ derived from osmometer measurement method could further rapidly streamline rankings of species’ iso/anisohydric stringency (Bartlett et al., 2012b).

The TLP is controlled by the accumulation of actively osmotic solutes (Kozlowski and Pallardy, 2002). A meta-analysis study found that Ψ_100_ was the main determinant of TLP (Bartlett et al., 2012a). In addition to Ψ_100_, the TLP is always related to the leaf modulus of elasticity (*ϵ*) (Meinzer et al., 2014). More negative Ψ_100_ and greater *ϵ* can contribute to the turgor maintenance as water content and water potential declines, which can maintain leaf physiological activity by maintaining protoplast volume, thus extending stomatal opening and photosynthesis with drying conditions (Meinzer et al., 2014; Johnson et al., 2018). Because stomata generally close around the TLP, stomata rarely closing at very negative water potential may reflect the finiteness of osmotic adjustment and the inability of foliage to maintain turgor at very negative Ψ_leaf_ (Bartlett et al., 2012a; Henry et al., 2019). Therefore, the position of species along the isohydric to anisohydric continuum would be scaled with species-specific variation in foliage osmotic properties (Meinzer et al., 2016; Hartmann et al., 2021).

The contribution of highly negative osmotic potentials to maintaining turgor is equivalent to more investment in high concentrations of compatible solutes (Johnson et al., 2018; Hartmann et al., 2021). The leaves of isohydric species would not undergo osmoregulation to maintain turgor during drought, which would decrease energy costs related to solute accumulation (Meinzer et al., 2014; Hartmann et al., 2021). However, excessive solute accumulation may result in the deleterious effects on leaf protein activity, which lead to a physiological limit to stomata opening, so osmotic potential is predicted to below the values of Ψ_close_ (Martin-StPaul et al., 2017).

Stomatal conductance is directly linked to leaf hydraulic conductance (K_leaf_), and stomatal closure have been shown to be related to a loss of K_leaf_, aiming to prevent leaf desiccation (Brodribb and Holbrook, 2003; Woodruff et al., 2015). The coordination between Ψ_leaf50_ (Ψ_leaf_ at 50% loss of conductivity) and TLP suggests that decline in K_leaf_ under drought should partially be a result of mesophyll cells losing turgor and shrinking (Scoffoni et al., 2014). Species with more negative TLP undergo less cell shrinkage and have slower declines in K_leaf_ during drought (Bartlett et al., 2016). Therefore, the K_leaf_ of more anisohydric species may be less vulnerable to drought (Nardini and Luglio, 2014), and more isohydric species were found to have lower hydraulic capacitance of leaves but higher K_leaf_ (Fu et al., 2019). Water storage through capacitance is also a factor in Ψ_close_ (Hammond and Adams, 2019). Nevertheless, there is no consistent conclusion on the relationship between Ψ_close_ and maximum G_s_ (Meinzer et al., 2017; Henry et al., 2019). Foliar water uptake was found to be an important water acquisition mechanism that can mitigate water deficits in some tree species, but the role of foliar water uptake on stomatal regulation remains unknown (Eller et al., 2013).

#### Structural investment

**Figure 2:**
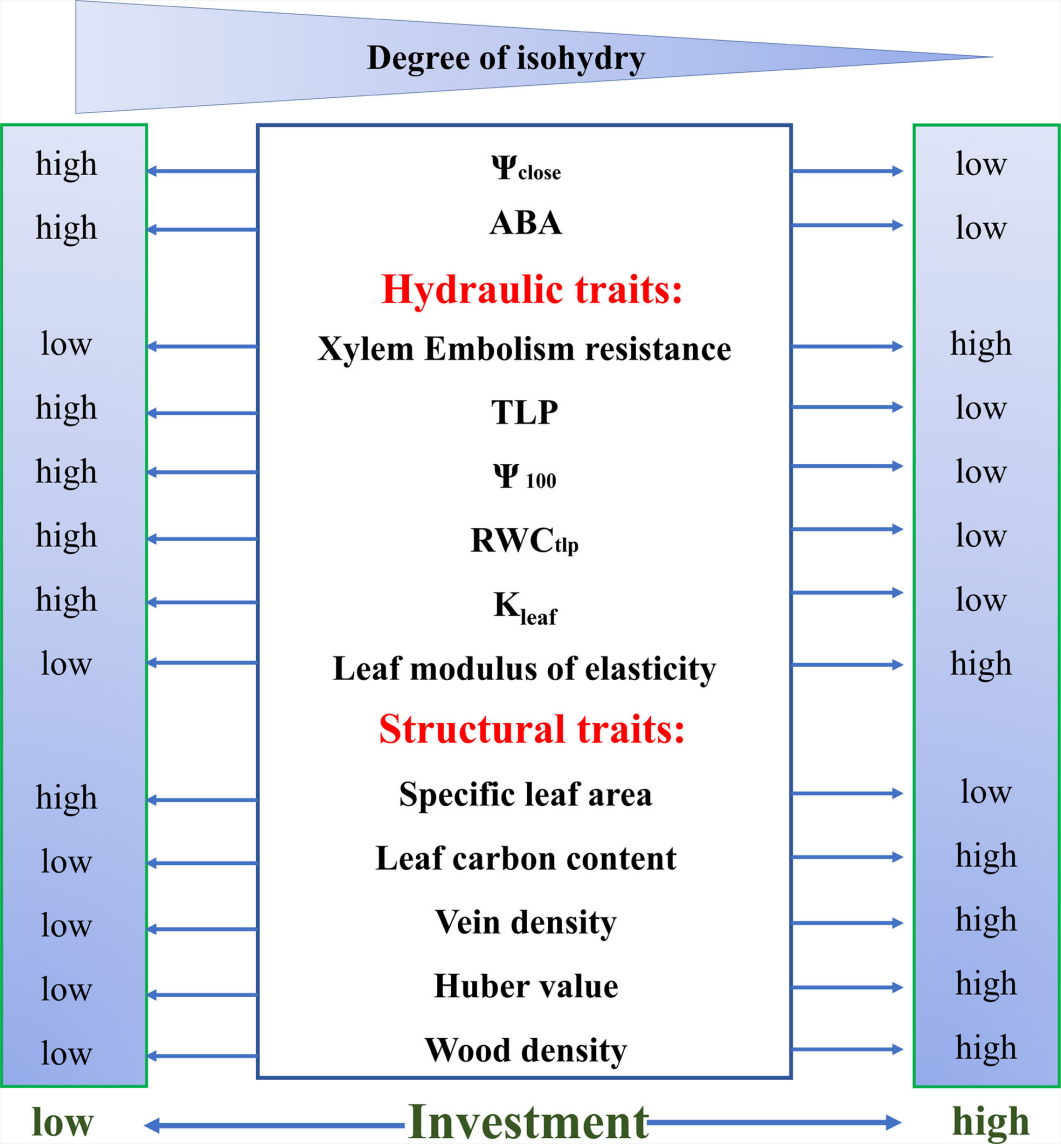
The relationships between the iso/anisohydric behavior and functional traits of leaves and branches, which show how these traits vary with the Ψ_close_. The anisohydric species show stronger stress resistance, with greater investment in stems and leaves than the isohydric species to maintain stomatal opening under drought conditions. Ψ_close_ (water potential at stomatal closure), TLP (turgor loss point), Ψ_100_ (osmotic potential at full turgor), RWC_tlp_ (relative water content at TLP), K_leaf_ (leaf hydraulic conductance). This figure is changed from Chen et al. (2021).

Stomatal behavior is regulated not only by water potential but also by structural adaptations that impact the demand and supply for water. Structural traits of leaf or stem such as vein density, specific leaf area (SLA), and wood density are essential to the exploring differential stomatal behaviors among species, which show the tradeoff between investment and stress tolerance (**Figure 2**).

Water flows from the petiole through leaf conduits that form a network of veins (McKown et al., 2010). However, vein ramification is costly in terms of material investment. Vein density was found to be significantly positively correlated with leaf carbon content and the degree of iso/anisohydry, which indicated that anisohydric tree species invested more carbon in leaves than did isohydric species (Chen et al., 2021b).

Specific leaf area (SLA) is the leaf area per unit of dry-mass investment, indicating high SLA requires less investment per unit area. More anisohydric plant species tend to have lower SLA (Pivovaroff et al., 2018; Chen et al., 2021b), and Ψ_close_ was found to be significantly related to leaf carbon content (Chen et al., 2021b). Wood density is an integrative trait and a direct manifestation of investment in wood, which was found to be correlated with the stringency of iso/anisohydry (Klein, 2014; Fu and Meinzer, 2019; Fu et al., 2019). Therefore, it can be concluded that the anisohydric species showed greater investment in foliage and stem than the isohydric species (Chen et al., 2021b; **Figure 2**).

### Variability of stomatal behavior

Seasonal adjustment in TLP was observed in lianas (Maréchaux et al., 2017), and leaf-level osmotic adjustment was observed in long-term drought experiment (Binks et al., 2016). Adjustments in *ϵ* have been also documented in the season (Meinzer et al., 2014). A more anisoyhdric species was observed that adjusted TLP during drought, whereas a co-occurring more isohydric species did not (Nolan et al., 2017). Meinzer et al. (2014) observed that the more anisohydric species had more negative TLP and Ψ_100_ with decreasing water availability, but TLP and Ψ_100_ in the more isohydric species did not change. In a anisohydric desert shrub, *Larrea tridentata*, TLP was observed to vary by up to 2 MPa over short periods (Meinzer et al., 1988; Johnson et al., 2018).

Anisohydric species may have to possess the plasticity in leaf P-V traits to compensate that their relatively limited stomatal regulation of maintaining stable leaf water potential (Meinzer et al., 2014; Johnson et al., 2018). In addition to P-V traits, the Ψ_leaf50_ of ansiohydric species also alters during the driest part of the summer (Johnson et al., 2018). Thus, it is reasonable to speculate that anisohydric species may have greater capacities to alter leaf biophysical properties, and individuals of some species may switch between isohydric and anisohydric behavior within species depending on developmental stages and environmental conditions (Meinzer et al., 2014; Meinzer et al., 2016; Johnson et al., 2018).

## Xylem embolism resistance

During a paleoclimatic crisis, hydraulic systems evolved under selective pressure to be highly resistant to embolism as increasing drought levels (Larter et al., 2017; Martin-StPaul et al., 2017), and species have evolved broad differences in embolism resistance under this strong selection (Choat et al., 2012).

Xylem safety threshold is the point in which a tree starts losing control over its hydraulic system. When the water potential is out of the safety threshold, continuing residual water loss can no longer be compensated by water uptake from the soil. Therefore, embolism resistance is one of the key traits responsible for predicting the drought tolerance of tree species (Brodribb and Cochard, 2009; Anderegg et al., 2016; Choat et al., 2018; Brodribb et al., 2020).

P_50_ is often used to characterize the xylem safety of trees (Brodribb and Cochard, 2009; Choat et al., 2012). When water potential is below P_50_, the water transport function will be markedly impaired (Choat et al., 2012; Choat et al., 2018; Arend et al., 2021). The hydraulic system of plants has evolved to the level of embolism resistance reaching P_50_ down to -18.8 MPa (Larter et al., 2015); and the most vulnerable species is found in tropical forest, whose P_50_ is as high as -0.18 MPa (Maherali et al., 2004).

Angiosperms exhibited higher intraspecific variation than gymnosperms in P_50_ (Anderegg, 2015). Higher angiosperm variability in P_50_ ought to result from anatomical differences between gymnosperms and angiosperms (Johnson et al., 2012; Anderegg, 2015). In addition, P_50_ is not the hydraulic safety threshold of all woody plants. Angiosperm species may be unable to recover if water potential dropped below P_88_ (the water potential inducing 88% xylem embolism), and P_50_ is considered more likely to be the hydraulic safety threshold of gymnosperms species (Urli et al., 2013). Environmental conditions and developmental stages may also affect the threshold to some degree. A recent field research of adult Norway spruce showed that processes leading to a rapid deterioration of tree hydraulic status and tree mortality occur in before P_50_ is reached (Arend et al., 2021; Li et al., 2022), suggesting that the vulnerability of some species to hydraulic failure may differ from previously expectations under different conditions.

The study of xylem embolism has a long history (Dixon and Joly, 1895; Ewart, 1906), and extensive research have been done in this field, including research methods (Cochard et al., 2013; Chen et al., 2021a). Thus, in this review, we do not dwell on the previous common description and only briefly describe the determinants of xylem embolism resistance from the perspective of anatomy.

### The determinants of xylem embolism resistance

Embolism occurs as ‘air-seeding’ enters the conduits through the pit pores, and pit area hypothesis proposes that the probability of embolism propagation in conduits increases with the total pit area of vessels (Tyree and Zimmermann, 2002; Wheeler et al., 2005). Larger vessels are more likely to have larger total pit area, so larger vessels may be more vulnerable to embolism (Jacobsen et al., 2007). Within species, smaller conduits were found to tend to be less vulnerable to embolism (Cai and Tyree, 2010; Fichot et al., 2015). However, the lack of comprehensive knowledge on the conduit size and pit traits further limits the understanding of the relationship between conduit dimensions and drought-induced embolism resistance (Cardoso et al., 2020). The porosity of pit membranes seems to be more important because pit membrane prevents bubble propagation (Tyree and Zimmermann, 2002). Thus, the importance of conduit size for embolism resistance remains a debate subject.

Pit membranes develop from the cell wall made of multiple cellulose layers, pectins and structural proteins, with a thickness between 140 nm and 1180 nm and including between 4 and 30 layers (Fichot et al., 2015; Kaack et al., 2021). The three-dimensional structure, hydration of pit membranes and their chemical composition play a key role in the resistance to embolism spread (Cardoso et al., 2020; Kaack et al., 2021). Perfusion experiments showed that cellulose and pectin are main components of pit membrane determining embolism resistance (Fichot et al., 2015).

**Figure 3:**
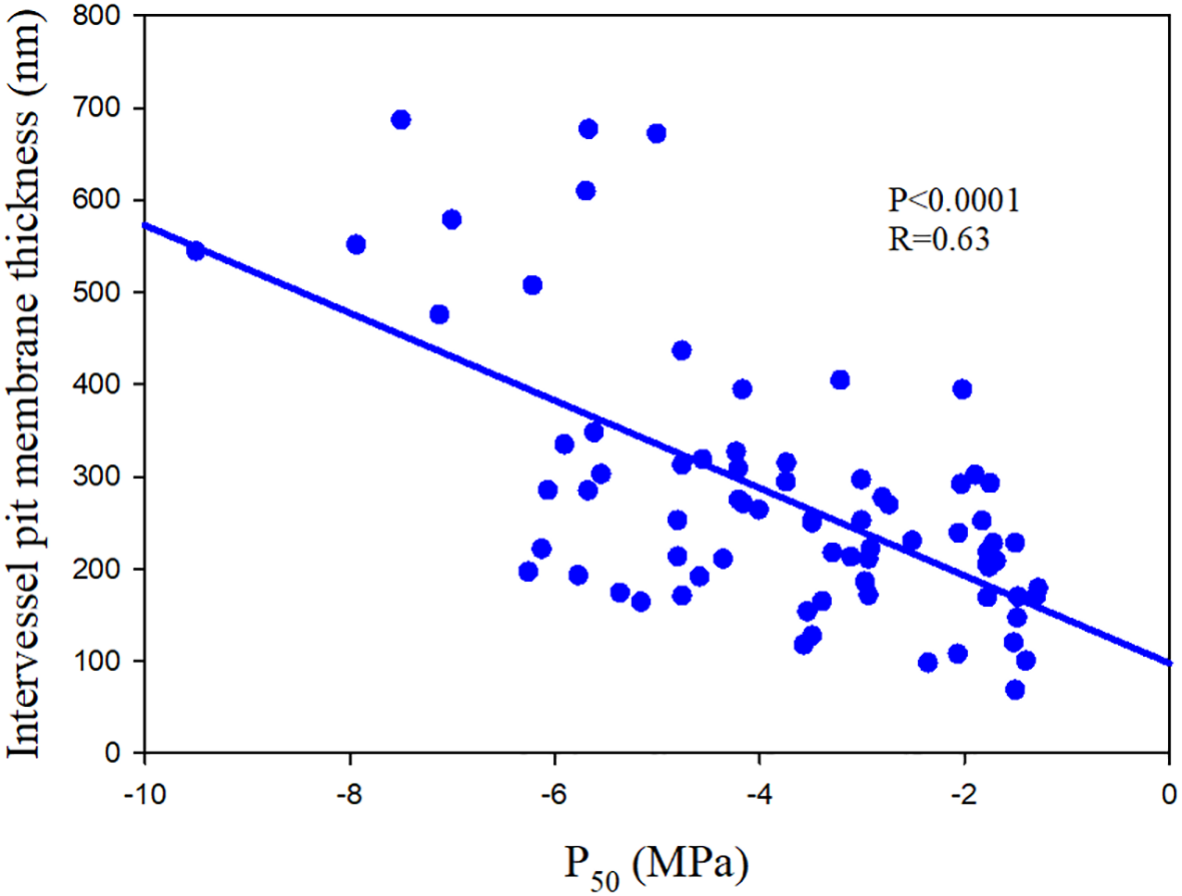
The relationship between water potential at which 50% loss of hydraulic conductance (P_50_) and intervessel pit membrane thickness of 75 angiosperm speices. The data comes from Scholz et al. (2013); Kaack et al. (2021) and Li et al. (2016).

Embolism spreading is related to the porosity of pit membrane, smaller pores tending to increase resistance (Choat et al., 2008; Fichot et al., 2015). Therefore, vulnerability to air-seeding is associated with the pit area, a bigger pit area implying an increased likelihood of having larger pores (Hacke et al., 2006). Thicker pit membrane is related to reduced porosity and therefore increasing resistance to embolism (Fichot et al., 2015; **Figure 3**). This link between embolism resistance and pit membrane thickness is also valid at the intrageneric (Scholz et al., 2013) and intraspecific level (Schuldt et al., 2016). Variation in pit membrane thickness is largely influenced by the number of microfibril layers, with thin pit membranes consisting of less microfibril layers than thick pit membranes (Kaack et al., 2021).

Species with thicker conduits walls have less porous and thicker pit membranes and consequent stronger embolism resistance (Li et al., 2016). Thick conduit walls also facilitate strong wall reinforcement (conduit thickness/span), which would against deflection and even implosion of conduit walls and possible injury to pit membranes (Hacke and Sperry, 2001; Hacke et al., 2001). An extensive and supportive fiber matrix is also associated with embolism resistance. It was proposed that small diameter fibers can result in dense wood and provide mechanical strength, which facilitate resisting embolism (Jacobsen et al., 2005; Mccullon et al., 2012; Blackman et al., 2019; Chen et al., 2020). Some studies also found that the positive relationship between fiber wall thickness and embolism resistance may be caused by lignification and wood density of xylem (Jacobsen et al., 2005; Awad et al., 2012; Fichot et al., 2015; Torres-Ruiz et al., 2017). Moreover, vasicentric tracheids and fiber–tracheids could act as water reservoirs, thereby increasing tissue capacitance, which are associated with lower cavitation vulnerability (Barotto et al., 2016).

**Figure 4:**
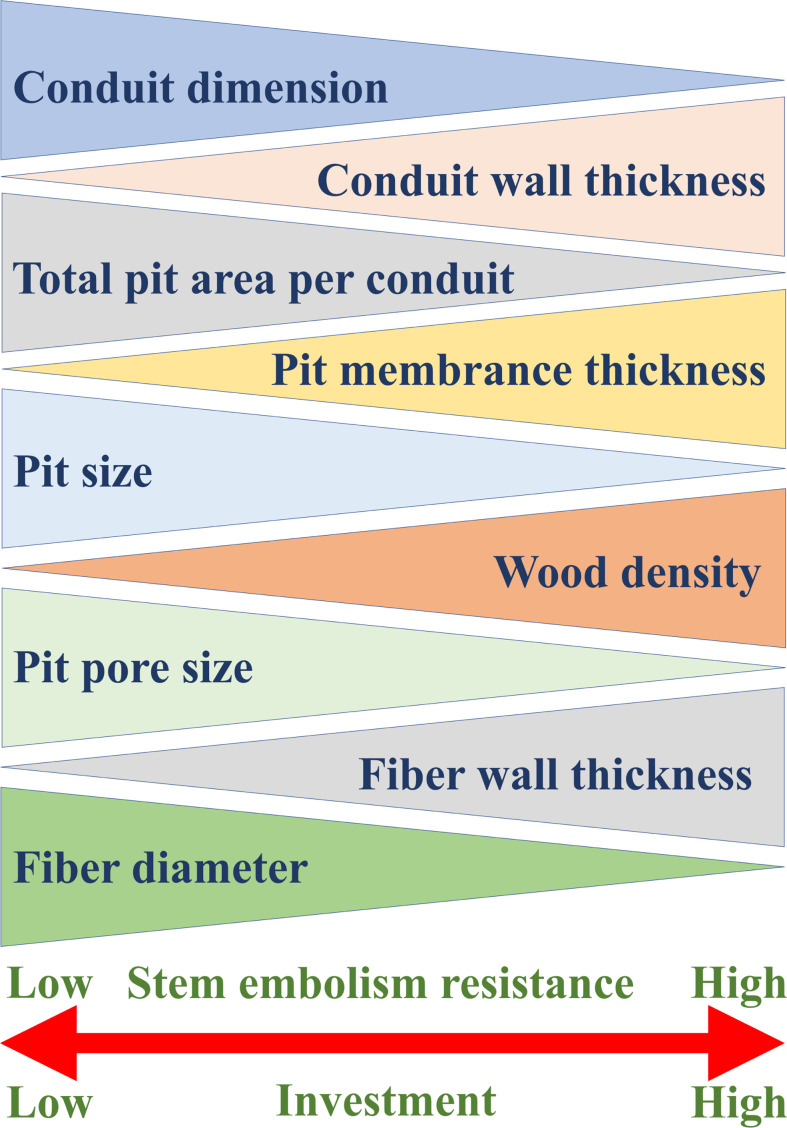
General xylem traits that determine stem embolism resistance and the relationships between these traits and stem embolism resistance. The thicker end of each triangle represents the higher value of the corresponding trait, and the xylem with stronger embolism resistance has greater construction investment.

Embolism-resistant traits, such as thick pit membrane, thick conduit wall, high wood density and high fiber-wall area, all are costly in terms of carbon investment, highlighting the relationship between stress resistance and carbon investment (**Figure 4**).

### Variability of embolism resistance

In general, the intra-specific P_50_ varied with more negative P_50_ values found in more xeric environments over space (Anderegg, 2015), and P_50_ values shifted toward more negative in response to drought stress (Wortemann et al., 2011). However, a few studies revealed that individuals exposed to drought stress were more vulnerable to embolism (Nardini and Luglio, 2014; Savi et al., 2015; Trugman et al., 2021). The explanation of this phenomenon is ‘cavitation fatigue’ for plants experienced chronic or extreme water stress (Hacke et al., 2001; Nardini and Luglio, 2014). Increased vulnerability to cavitation of the species, *Quercus ilex* L. (holm oak), exposed to long-lasting growing at urban sites under impervious pavements or 6-yr partial rainfall exclusion, might be explained as the hydraulic damage caused by cavitation fatigue (Limousin et al., 2010; Savi et al., 2015). In addition, cavitation fatigue has been also applied to explain the differences of embolism resistance between sapwood and heartwood (Fichot et al., 2015). A previous study demonstrated that conduits of the outer ring were still functional, whereas conduits of the older xylem were almost all embolized (Sperry et al., 1991).

The nutritional environment may also affect intra-specific variation of embolism resistance. High N availability tends to increase porosity of the pit membranes of vessel, and therefore, N-fertilized individuals have been shown to be more vulnerable to embolism (Plavcová and Hacke, 2012; Plavcová et al., 2013; Fichot et al., 2015). Additional P supply tends to increase embolism resistance, especially at high N concentrations (Harvey and Van Den Driessche, 1997; Hevia et al., 2019). As the xylem of species/genotypes more resistant to embolism needs more carbon investment and the carbon resources of shaded plants are limited, shaded individuals tend to be more vulnerable to cavitation (Schoonmaker et al., 2010; Plavcová et al., 2011). In addition, phloem conductance and water storage affect the recovery of embolism (Hammond and Adams, 2019). Nevertheless, intra-specific variation of vulnerability to embolism should be small, and a study of one widespread pine species (*Pinus pinaster*) showed low variation in genes of P_50_ across different populations (Lamy et al., 2014).

## Root properties

Plant root system plays a significant role in plant growth by exploiting soil resources through the uptake of water and nutrients (Wasaya et al., 2018). In general, resource absorption is undertaken by roots of terminal branch orders (usually the first- and second-order roots), whereas roots of the higher branch orders perform other functions, such as anchorage and storage (McCormack et al., 2015). Trees within the same forest are equipped with different rooting depths, so available water of trees varies with depth. In general, deep roots, i.e. deep-water access, may mitigate drought-induced mortality by limiting exposure to water stress. In a tropical forest, species exposure to drought stress exponentially declined with deeper root depth, which was relevant to drought resistance and resilience (Chitra-Tarak et al., 2021).

Root traits such as fine root diameter, specific root length, root angle, root length density and root hydraulic traits are considered to be linked with root water acquisition, water use efficiency, drought tolerance and access to nutrient, etc., which are important for understanding plant growth, survival and productivity (Nelson and Oliver, 2017; Wasaya et al., 2018; Tracy et al., 2020). The anatomical traits for the adaptations to soil water content can be determined by indices of the ratio of the root tissue areas (i.e. cortex to stele ratio, xylem to stele ratio) (Yamauchi et al., 2020). Root hairs are subcellular protrusions of the root epidermis, which extend into the soil and can improve the water and nutrient capture (Lynch, 2019). Importantly, root hydraulic traits are directly related to the capabilities and strategies of root organ and even whole plant to adapt to drought. Losing water from the root cells causes dehydrating roots to shrink, physically reduces hydraulic conductance at the soil-root interface, and causes roots to lose turgor which is crucial to root growth (Bartlett et al., 2021b). Roots with a more negative Ψ_100_ would exhibit a higher cell solute concentration and consequently more negative Ψ_TLP_, and roots with a greater RWC_TLP_ would retain more water at Ψ_TLP_ (Bartlett et al., 2021b). A lower root capacitance indicates that the roots retain more volume *via* less cell shrinkage, and thus maintain greater contact with the soil (Bartlett et al., 2021b). Hydraulic redistribution describes the passive flux of water through roots, for example from moist to dry soil layers (Hafner et al., 2020). Osmotic adjustment would strengthen the water potential gradient driving hydraulic redistribution, and higher hydraulic conductivities and larger conduits are also found to be related to higher hydraulic redistribution quantity (Hafner et al., 2020; Bartlett et al., 2021b). Root cortical lacunae usually precede root xylem embolism under drought stress, and embolism usually appears first in the fine roots and then in older, coarse roots (Cuneo et al., 2016). However, turgor and embolism recovery of roots occurs quickly upon exposure to water (c. 40 min-4 h) by absorbing inorganic ions and water (Shabala and Lew, 2002; Cuneo et al., 2018). Although the research on root hydraulic characteristics has made great progress, the research is still relatively backward compared with the aboveground part. Due to the inaccessibility of roots, new methods for root assaying need to be developed in the future (Nelson and Oliver, 2017).

## Coordination of stomatal regulation, xylem embolism resistance and root properties

**Figure 5:**
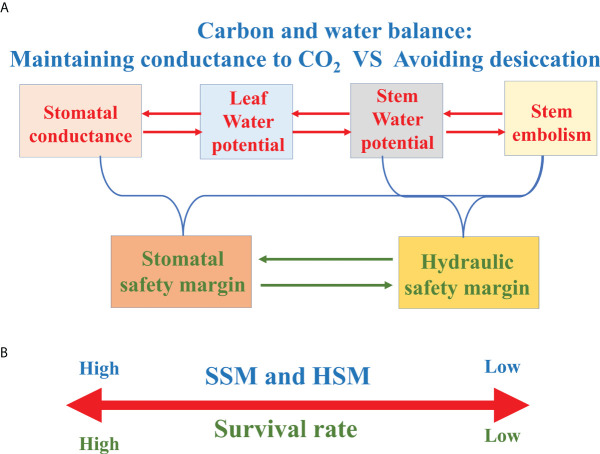
**(A)** Frame diagram characterizing the relationships between stomatal conductance, leaf/stem water potential and stem xylem embolism. **(B)** The survival rate of tree species with greater hydraulic safety margin (HSM) or stomatal safety margin (SSM) is higher under drought conditions.

**Figure 6:**
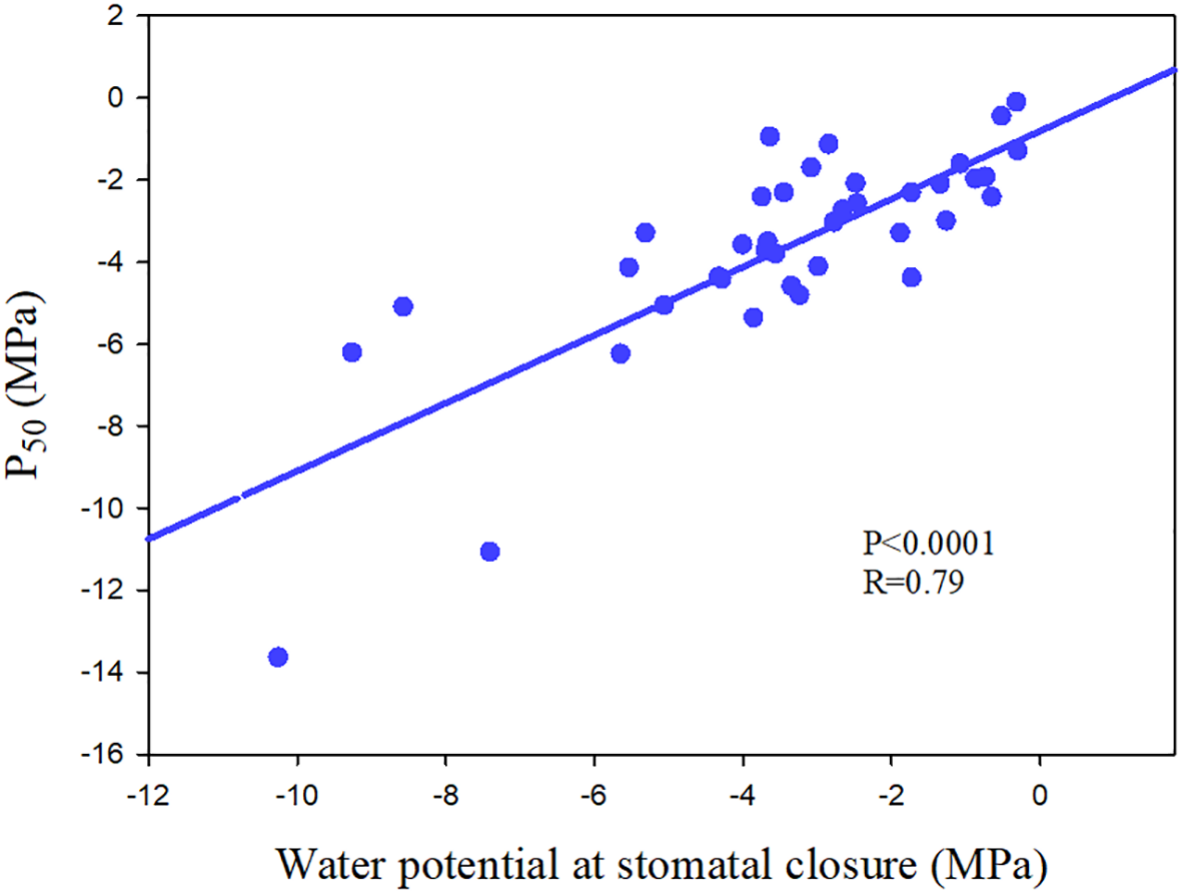
The relationship between water potential at stomatal closure and water potential at which 50% loss of hydraulic conductance (P_50_) of 39 angiosperm speices. The data comes from Pivovaroff et al. (2018); Chen et al. (2019a) and Li et al. (2015).

The ability of maintaining high surface conductance to CO_2_ while avoiding desiccation is pivotal for survival in land plants. While reducing leaf G_s_ for reducing water loss has the cost of constraining photosynthetic carbon sequestration (Cowan and Farquhar, 1977). Therefore, the functional properties of the plant hydraulic system are integral to carbon and water balance (Drake et al., 2015; **Figure 5A**), and the ability of maximizing carbon gain and minimizing water loss has served as a powerful selective force on the evolution of functional and structural adaptations to drought in plants (Ambrose et al., 2009). Strong correlations between iso-anisohydric behavior and stem traits were observed in recent studies, highlighting the coordination of stomatal regulation and xylem function (Chen et al., 2021b; **Figure 6**). Many species with similar root syndromes display contrasting aboveground traits, which highlights the importance of including belowground organs to the whole-plant trait integration (Carmona et al., 2021). However, researches on the relationship between stem and leaf traits and root properties are still scarce, thus there is an important research task in the future in order to fully understand the coordination of tree water relations.

### The correlation of stomatal behavior with xylem properties

The stomatal closure for preventing Ψ_leaf_ from falling below a set minimum value facilitates keeping the stem xylem from experiencing more serious embolism (Meinzer et al., 2014), and xylem embolism may also produce rapid hydraulic signals for initiating the stomatal response (Salleo et al., 2000). The hydraulic message triggered by embolism including ABA and pH changes in the xylem sap that travels to the shoots and contributes to the regulation of G_s_, together with the direct effect of xylem water potential (Salleo et al., 2000; Tardieu, 2016). In other words, stomatal regulation and stem embolism are mutually linked by a negative feedback mechanism (Salleo et al., 2000).

The iso/anisohydric strategies combined with the corresponding xylem traits reflect the drought avoidance strategy and drought tolerance strategy. Drought avoidance and drought tolerance are two divergent strategies promoting plants adaptation to drought (Oliveira et al., 2021). The continuum of stomatal strategy from isohydry to anisohydric basically coincides with that from drought avoidance to drought tolerance. Drought-avoiding species close stomata rapidly responding to increased water deficit, and even directly shed leaves, which constrains carbon assimilation during dry periods; such drought avoiders with low wood density and high hydraulic capacitance appear to avoid xylem embolism *via* releasing of stored water to decrease Ψ_leaf_ fluctuations (Brodribb et al., 2014; Oliveira et al., 2021). On the contrary, species of drought tolerance with denser wood and low capacitance appear to rely on xylem structure to resist embolism and could maintain foliage over dry periods (Fu et al., 2019). Therefore, drought tolerators can sustain relatively photosynthesis, albeit at the cost of enhanced investment in structural reinforcement of embolism-resistant xylem and in osmoregulation to sustain leaf turgor (Meinzer et al., 2016). In general, anisohydric species appear to occupy more drought-prone habitats compared with isohydric species (McDowell et al., 2008).

### Hydraulic safety margin

The concept of HSM is generally described as the difference between a critical point (usually P_50_ or P_88_) on the vulnerability curve to embolism and the minimum seasonal xylem water potential (Ψ_min_). In view of the significant correlation between the Ψ_min_ and Ψ_close_, Ψ_min_ is often assumed to provide a measure of stomatal regulation of water potential (Pivovaroff et al., 2018; Chen et al., 2019a). As woody plants tend to maximize carbon sequestration relative to the investments of constructing or maintaining hydraulic support for foliage, woody species routinely operate at the catastrophic brink of xylem failure, highlighting the importance of the margin between xylem water potential and P_50_ or P_88_ (Tyree and Sperry, 1988; Bond and Kavanagh, 1999). Therefore, large positive HSM implies a relatively conservative response and small HSM (or even negative HSM) suggests a hydraulically risky response in the control of plant water relations (Skelton et al., 2015; Trugman et al., 2021). Although drought-induced tree death is complex and involves many physiological traits and processes, numerous previous studies showed that HSM was good (even only) predictor of mortality or branch dieback for tree species or shrub species (Adams et al., 2017; Chen et al., 2019a; **Figure 5B**).

According to the definition of HSM, HSM is determined by both Ψ_min_ and embolism resistance. Although embolism resistance is strongly associated with the Ψ_min_ across species, more embolism-resistant species have larger hydraulic safety margin than sensitive ones (Markesteijn and Poorter, 2011; Fu and Meinzer, 2019). HSM also affects species-specific distribution, and species that inhabit more arid environments usually possess larger HSM than those occupying wetter ones (Garzón et al., 2018). In addition, there appears to be a tendency for coniferous species to have larger HSMs compared with most angiosperm species (Choat et al., 2012), with Mediterranean climate angiosperms being a potential exception (Meinzer and McCulloh, 2013). Pioneer species usually operate lower HSM than shade-tolerant species which usually have more conservative strategies, and strong differences in HSM were also found between co-occurring deciduous and evergreen species (Markesteijn and Poorter, 2011). To offset a shorter growing season, deciduous species generally maximize photosynthetic carbon intake in the growth season (Wright et al., 2004; Ishida et al., 2006), and would thus operate a greater danger in hydraulic failure to maximize productivity in short growth season (Markesteijn and Poorter, 2011).

Unfortunately, for acquiring HSM, datasets of Ψ_min_ in field conditions are typically patchy because of the laborious onsite measurement techniques (Breshears et al., 2009; Choat et al., 2018), and long-term measurement of Ψ_min_ across seasonal scales is very time consuming. In addition, the hydraulic resistance in leaves is dynamic over a day timescale, which would lead to pronounced transpiration-induced disequilibrium between leaf and stem water potential in transpiring shoots (Meinzer et al., 2009). Therefore, stem xylem water potential of field grown plants must be measured on non-transpiring (shading treatment) leaves or shoot tips attached to transpiring shoots (Meinzer et al., 2009), which also increases the difficulty of measuring the Ψ_min_ especially in tall trees.

### Stomatal safety margin

Stomatal safety margin (SSM) refers to the difference between Ψ_close_ and the water potential causing xylem dysfunction (usually P_50_). Compared with HSM, SSM uses Ψ_close_ instead of Ψ_min_, which offsets the difficulty of obtaining the Ψ_min_ data mentioned above. In addition, SSM more directly integrates the “safety” of stomatal response to water potential and xylem to drought-induced embolism (Skelton et al., 2015; **Figure 5A**).

Stomatal regulation preventing leaf water potential from falling below a set minimum value facilitates constraining excessive loss of stem hydraulic conductivity. This is in agreement with the hydraulic segmentation hypothesis, i.e., more distal components such as foliage protect stems by earlier embolism under drought (Tyree and Ewers, 1991; Nolf et al., 2015). Large positive SSM represent that leaves stomatal closure occurs before stem severe embolism, whereas negative SSM indicate stomatal closure subsequent to P_50_ (Skelton et al., 2015).

Ψ_close_ varied from -0.655 MPa to -5.54 MPa (Martin-StPaul et al., 2017; Chen et al., 2019a), with the outlier about -10 MPa in a chaparral species in Mediterranean-type climate (Pivovaroff et al., 2018), spanning a range of variation about one-third that for embolism resistance (P_50_, -0.18 MPa to -18.8 MPa) (Maherali et al., 2004; Larter et al., 2015). Meta-analysis showed that most species have Ψ_close_ values that are higher than their P_50_ (negative value) (Martin-StPaul et al., 2017; **Figure 6**). However, in the published data, it is found that the stomatal closure of a little tree species occurs after the occurrence of large xylem embolism, and such outliers are easy to die during drought (Skelton et al., 2015; Pivovaroff et al., 2018; Chen et al., 2019a; Chen et al., 2019b). In addition, there are species with strong embolism-resistant stems ultimately experience high mortality, which may result from the terrible coordination between stomatal regulation and xylem embolism resistance (Anderegg et al., 2019). There are clear evidences of partial and complete mortality correlated with hydraulic failure in both isohydric and anisohydric plants (McDowell, 2011), which further highlight the importance of coordination between stomatal regulation and xylem embolism resistance.

After stomatal closure, it still shows a decline of water potential, which is likely driven by cuticular and residual stomatal conductance. Nonetheless, the time of desiccation of species with wider SSM was longer (Blackman et al., 2019; Cardoso et al., 2020), and species that closing stomata relatively late also assume risky safety margin (Skelton et al., 2015). In general, SSM increased continuously with increasing embolism resistance (Martin-StPaul et al., 2017), and SSM is correlated with the HSM (Chen et al., 2019a). Most importantly, merging stomatal regulation strategies with xylem embolism resistance strategies contributes to a more comprehensive framework to manifest plant adaptation to drought (Skelton et al., 2015).

### Coordination between leaf and stem xylem traits and root properties

The dialogue between leaves, stems and roots is important in water uptake, transport and utilization. Water uptake occurs when the water potential of root system is higher than leaf water potential, thus establishing a gradient for water flux. In general, there is a linkage between the circadian oscillations in root hydraulic conductance and the daily variations in the transpiration, with significant consequences on water uptake (Tardieu et al., 2017; Trugman et al., 2021).

In terms of coordination between roots and leaf stomata, varieties in soil-plant hydraulic conductance would drive stomatal closure (Cai et al., 2022). Root-produced signaling may also affect stomatal conductance, and split root experiments have the potential to enable the investigation of the water uptake pattern of root systems (Koebernick et al., 2015). Previous studies found that when the dry part of the root system is at an intermediate soil water status, signal output from the entire root system is maximized, such that further soil drying actually decreases root-to-shoot signaling (Dodd et al., 2010). Alternatively, root capacitance might also impact gas exchange. Roots with a lower capacitance, which indicates that roots retain greater volume as water potentials decline, tend to maintain greater gas exchange through smaller declines in root volume and less ABA production (Bartlett et al., 2021b).

In terms of coordination between roots and stem hydraulic traits, there may be a trade-off between root depth and stem xylem embolism resistance. A study found that species exposure to drought stress declined with deeper root depth indicating that trees compensate for drought stress-related mortality risk through deep-water access, whereas species with deeper root had lower stem xylem embolism resistance and narrower stem hydraulic safety margins (Chitra-Tarak et al., 2021; Trugman et al., 2021). Species with investment in deep roots can access reliable deep-water resources, which ensures that hydraulic risk may not realize for them. Nevertheless, shallow-rooted species may tend to pay the cost of stem hydraulic safety, adapting for an environment in which hydraulic risk may be prone to occur, as extreme droughts usually occur in shallow soil layers (Chitra-Tarak et al., 2021). In addition, root systems have metabolic costs of respiration, another trade-off therefore may exist between the carbon cost of the root system and the growth of other organs (for example, stem) (Tardieu et al., 2017).

## Perspectives

As mentioned above, sound knowledge of tree hydraulic function is crucial to understand drought-induced tree mortality, and it is necessary to extend and integrate other functional traits and processes to establish a more comprehensive framework.

Stomatal regulation and xylem embolism resistance are likely to interact with other important traits and processes, e.g., leaf area, plant water storage, root properties, hydraulic segmentation, minimum bark conductance and metabolic status, in the plant responses to drought (Hammond and Adams, 2019; Trugman et al., 2021). We can take stomatal regulation and xylem embolism resistance as the core, expand other important traits, including the relationship between investment of matter allocation and stress resistance, and incorporate the thoughts of the ‘fast-slow’ plant economics spectrum to establish a more comprehensive combination of traits that reflects tree species’ response and adaptation to drought. In functionally diverse communities, an integrated traits combination may produce more diverse drought adaptation strategies (Martínez-Vilalta and Garcia-Forner, 2017) and will accurately improve our prediction of how plants to adapt to spatio-temporal changes in environment conditions, which facilitates to model drought-related tree mortality and changes in plant demography in the future (Rowland et al., 2021).

In addition, we propose four specific urgent issues as future research priorities:

(1) We should expand future works to not only quantify empirical thresholds for hydraulic failure and associated HSM or SSM but also understand species specific variations over time to reach the thresholds (P_50_, Ψ_close_, and so on), which requires further knowledge of a range of other traits such as rooting depth, phloem conductance and plant water storage, and so on (Hammond and Adams, 2019).

(2) There is uncertainty in the mechanisms about the refilling of embolized xylem and the point of no return along P_50_, Ψ_close_, HSM or SSM, which limits our ability to understand and predict the legacies of successive drought events. This uncertainty is also related to the lack of knowledge about the interdependence of hydraulic failure and carbon starvation. Increased embolism and associated xylem tensions would cause declining carbon gain, transport, utilization, and subsequent feedbacks by which declining NSC availability impacts embolism through refilling or water homeostasis, but studies in this area is still lacking (McDowell, 2011; Hammond and Adams, 2019).

(3) It is not fully understood how iso/anisohydric behavior, xylem embolism resistance and HSM or SSM are affected by the root elements, especially root depth and root capacitance. Tree species with different iso/anisohydric behaviors and HSM or SSM may have different investments in above-ground woody stem and below-ground root growth (Ambrose et al., 2015). In addition, it is not clear that how chemical messages originating from roots affect canopy stomatal regulation under field conditions, especially for tall trees (Tardieu, 2016), and how roots as storage organs compensate for aboveground death by resprouting.

(4) The increase of the air temperature is the main hallmark of global climate change and is coupled with droughts, hence directly or indirectly affecting drought adaptation/resistance/resilience strategies of tree species. Some species exhibited higher stomatal conductance and stomatal density in the wet season in warming experiment (Wu et al., 2020). However, there is a lack of research on the effect of long-term warming on iso/anisohydric regulation, stem xylem embolism resistance and HSM or SSM, and thus future research in this area should be carried out.

## Author contributions

ZC, XW and SRL conceived the ideas. ZC, SL, and SRL led the writing of the manuscript. ZC and XW revised the manuscript. All authors contributed to the article and gave final approval for publication.

## Funding

The research was supported by the Fundamental Research Funds of Chinese Academy of Forestry (CAFYBB2020QB009), the Ministry of Science and Technology of China for Key R&D Program (2021YFD2200405) and the National Natural Science Foundation of China (31800513).

## Acknowledgments

We would like to thank Baotianman Forest Ecosystem Research Station for the assistance in the previous research related to the ideas of this paper.

## Conflict of interest

The authors declare that the research was conducted in the absence of any commercial or financial relationships that could be construed as a potential conflict of interest.

## Publisher’s note

All claims expressed in this article are solely those of the authors and do not necessarily represent those of their affiliated organizations, or those of the publisher, the editors and the reviewers. Any product that may be evaluated in this article, or claim that may be made by its manufacturer, is not guaranteed or endorsed by the publisher.
